# Influence of Pore Size in Protein G'‐Grafted Mesoporous Silica Nanoparticles as a Serum Pretreatment System for In Vitro Allergy Diagnosis

**DOI:** 10.1002/adhm.202203321

**Published:** 2023-03-08

**Authors:** Juan L. Paris, Cristina Monío, Ana M. Pérez‐Moreno, Raquel Jurado‐Escobar, Gador Bogas, Tahía D. Fernández, María I. Montañez, Cristobalina Mayorga, María J. Torres

**Affiliations:** ^1^ Allergy Research Group Instituto de Investigación Biomédica de Málaga y Plataforma en Nanomedicina‐IBIMA Plataforma BIONAND. RICORS “Enfermedades inflamatorias” Málaga 29590 Spain; ^2^ Facultad de Ciencias Universidad de Málaga Málaga 29010 Spain; ^3^ Allergy Unit Hospital Regional Universitario de Málaga‐HRUM Málaga 29009 Spain; ^4^ Departamento de Biología Celular Genética y Fisiología Facultad de Ciencias Universidad de Málaga Málaga 29010 Spain; ^5^ Departamento de Química Orgánica Universidad de Málaga‐UMA Málaga 29010 Spain; ^6^ Departmento de Medicina Universidad de Málaga‐UMA Málaga 29010 Spain

**Keywords:** allergies, immunoglobulin, in vitro diagnostics, mesoporous silica nanoparticles

## Abstract

Particles with the capacity to bind to immunoglobulin G (IgG) can be used for the purification of IgG or to process clinical samples for diagnostic purposes. For in vitro allergy diagnosis, the high IgG levels in serum can interfere with the detection of allergen‐specific IgE, the main diagnostic biomarker. Although commercially available, current materials present a low IgG capture capacity at large IgG concentrations or require complex protocols, preventing their use in the clinic. In this work, mesoporous silica nanoparticles are prepared with different pore sizes, to which IgG‐binding protein G’ is grafted. It is found that for one particular optimal pore size, the IgG capture capacity of the material is greatly enhanced. The capacity of this material to efficiently capture human IgG in a selective way (compared to IgE) is demonstrated in both solutions of known IgG concentrations as well as in complex samples, like serum, from healthy controls and allergic patients using a simple and fast incubation protocol. Interestingly, IgG removal using the best‐performing material enhances in vitro IgE detection in sera from patients allergic to amoxicillin. These results highlight the great translation potential of this strategy to the clinic in the context of in vitro allergy diagnosis.

## Graphical abstract

In this work, protein G’‐grafted mesoporous silica nanoparticles with different pore sizes are reported. The results show that mesopore size influences IgG capture capacity, being ≈12 nm the optimal pore size. With this material, an efficient and specific IgG removal protocol is presented for human serum, which enhances detection of amoxicillin‐specific IgE by Phadia ImmunoCAP.

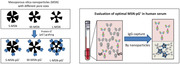

## 1 Introduction

In vitro detection of immunoglobulins (Ig) in serum is a widely used strategy for the diagnosis of a multitude of pathologies, from infectious diseases to autoimmune and allergic diseases.^[^ ^1^ ^]^ In the case of allergy, most in vitro diagnostic techniques are based on the detection of allergen‐specific IgE as the main biomarker.^[^ ^2^ ^]^ This is because the most common mechanism of activation of an allergic response begins with recognition of the allergen by at least two adjacent IgE molecules that are bound to the high‐affinity receptor (Fc*ε*RI) on the cell membrane of mast cells and basophils. These allergen‐specific IgE can be detected bound to this effector cells membrane as well as soluble in serum, being IgE levels much lower than IgG (by far the most abundant immunoglobulin in serum). This is particularly critical for certain types of allergies such as drug allergy, where the amount of drug‐specific IgE present in serum is even lower and may not be detected with currently available techniques due to their low sensitivity.^[^ ^3^ ^]^ This forces the use of in vivo diagnostic methods (skin tests and mainly controlled exposure to the allergen or provocation), which not only involve a higher cost for the healthcare system, but also pose a risk for the patient, especially in cases of severe allergic reactions, since exposure to the allergen/drug during the diagnostic test can trigger an anaphylactic reaction.^[^ ^3^ ^]^ Therefore, there is an increasing need for improving the sensitivity of risk‐free in vitro tests such as immunoassays for evaluating allergic diseases.

Immunoassays for in vitro allergy diagnosis consist of a solid phase to which an allergenic structure is conjugated and incubated with the serum of patients with a suspected allergy.^[^ ^4^ ^]^ Subsequently, the use of an anti‐IgE secondary antibody (with radioactive or fluorescent labeling or an enzyme participating in a colorimetric enzyme) makes it possible to identify the presence of specific IgE against the allergen tested in the patient's serum.^[^ ^4^ ^]^ In this context, it is known that several factors limit the sensitivity of these diagnostic tests, deriving one of them from the high concentration of total IgG in serum (7–16 mg mL^−1^) compared to the amount of total IgE (about 10 000–50 000 times less concentrated in healthy individuals, and around 1000 times less concentrated even in highly atopic individuals).^[^ ^5^ ^]^ The presence of allergen‐specific IgG that occupies part of the allergenic structures present in the solid phase, could reduce the recognition sites available for binding of specific IgE, which is the analyte to be detected. Not only does the binding of allergen‐specific IgG limit sensitivity, but the presence of high amounts of IgG, even if it is not specific for the allergen, also limits the sensitivity of in vitro diagnostic allergy tests. Additionally, the popularization of multiplex allergy diagnostic methods (simultaneous detection of immunoglobulins against multiple allergens) worsens this situation, since false positives have been described due to the incorrect detection of allergen‐specific IgG (of unknown clinical utility) as if it were allergen‐specific IgE (which is the validated biomarker for diagnosis of allergic diseases).^[^ ^6^, ^7^ ^]^

**Figure 1:**
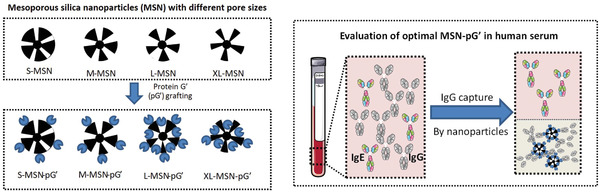
Schematic representation of the work.

For all these reasons, the use of sample pretreatment systems based on the removal of IgG from serum prior to its analysis by in vitro diagnostic allergy tests has been proposed.^[^ ^8^, ^9^ ^]^ To carry out this removal, commercial capture systems based on particles or columns that have conjugated a protein with selective binding capacity to the constant region of human IgG (and without IgE binding capacity) have been used. The two most common proteins with this capacity are protein A and protein G. Protein A has a high uptake capacity for various types of human IgG, but with limited binding capacity to IgG3 (which constitutes about 8% of total IgG in human serum).^[^ ^10^ ^]^ On the other hand, protein G has a broader binding profile to all types of human IgG (including IgG3), but it also possesses albumin recognition regions, which has led to the development of modified forms of the protein lacking this region (such as protein G'), to ensure specific binding of IgG without albumin uptake.^[^ ^10^ ^]^ As mentioned above, there are multiple commercial systems containing these proteins, and when these materials have been used as serum pretreatment methods, increased sensitivity in the detection of allergen‐specific IgE has been observed in both mouse and human samples.^[^ ^8^, ^9^ ^]^ These preliminary data would demonstrate the usefulness of a serum IgG removal system as a sample pretreatment method in the context of in vitro diagnosis of allergy by detection of allergen‐specific IgE. However, existing commercially available systems either require complex protocols and high incubation times or sample volumes,^[^ ^7^ ^]^ or have a low IgG uptake capacity (6–125 µg IgG mg^−1^ of material), requiring multiple cycles of serum treatment to achieve the required level of IgG removal.^[^ ^11^ ^]^ These limitations have prevented the translation of these strategies to routine clinical diagnostics. Commercially available products consist of a solid phase (in the form of nano‐ or micro‐particles in powder or suspension or within an affinity column) with protein A, protein G or a combination of both conjugated to the surface of the material. There are commercial products manufactured with particles of different compositions and sizes, and the effect of pore size in IgG capture has been studied for macroporous silica materials (pore size >50 nm).^[^ ^12^ ^]^ However, there are no commercial systems for IgG removal based on mesoporous particles (with pores in the range 2–50 nm), and the effect of mesopore size in IgG removal remains largely unexplored. Given that the size of the proteins used for IgG uptake is in the same size range as that of mesopores, our starting hypothesis is that there may be an optimal pore size that allows an enhanced anchoring of the protein to the nanoparticles in a conformation that boosts their performance. This hypothesis can be supported by data reported by other authors regarding the use of mesoporous nanoparticles for the release of protein antigens, where they observed that modification of the pore size of the particles leads to modification of the behavior of the particles for therapeutic application.^[^ ^13^ ^]^ Thus, in this work we report the preparation and characterization of protein G’‐grafted mesoporous silica nanoparticles (MSNs) with different well‐defined pore sizes in order to study the effect of mesopore size in their IgG capture capacity (**Figure** **1**). Once the best performing material was selected, the particles with the optimal pore size were evaluated in serum samples from both healthy controls and allergic patients to confirm the potential for their translation to the clinical setting in the context of in vitro allergen‐specific IgE detection for allergy diagnosis.

## 2 Results and Discussion

### 2.1 Synthesis and Characterization of MSNs

**Figure 2:**
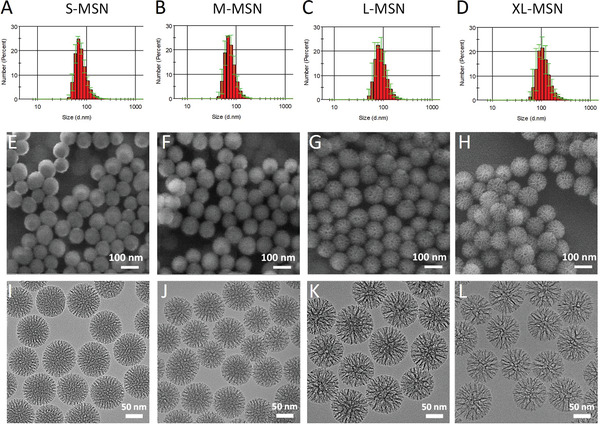
Characterization of prepared MSNs. A–D) Nanoparticle size distribution determined by DLS, middle‐H) SEM micrographs showing particle size and morphology and I–L) TEM micrographs showing particle size, morphology, and mesopore structure. Results correspond to S‐MSN (A,E,I), M‐MSN (B,F,J), L‐MSN (C,G,K), and XL‐MSN (D,H,L).

MSNs with four different pore sizes were prepared: small (S‐MSN), medium (M‐MSN), large (L‐MSN), and extra‐large (XL‐MSN). The correct preparation of the materials was confirmed by dynamic light scattering (DLS), scanning electron microscopy (SEM), transmission electron microscopy (TEM), and nitrogen adsorption. The DLS results showed monodisperse nanoparticles with sizes in the range of 70–100 nm in diameter for the four types of materials prepared (**Figure** **2**). SEM images confirmed the particle size and showed spherical morphology (Figure 1). TEM results confirmed both the particle size observed by DLS and the presence of pores with the desired size: pore S‐MSN < pore M‐MSN < pore L‐MSN < pore XL‐MSN (Figure 2). The pore size of each particle type was finally determined by nitrogen adsorption (**Table** **1**, Figure S1, Supporting Information), observing a pore size of 5.75 nm for S‐MSN, 8.53 nm for M‐MSN, 12.22 nm for L‐MSN and 14.23 nm for XL‐MSN. In all cases, particles with a large surface area (>400 m^2^ g^−1^) were obtained.

**Table 1:** Textural properties of prepared MSNs as determined by N_2_ adsorption

Sample	BET* surface area [m^2^ g^−1^]	Pore volume [cm^3^ g^−1^]	Pore width [nm]
S‐MSN	417.5	0.56	5.75
M‐MSN	455.3	0.8	8.53
L‐MSN	679.5	1.41	12.22
XL‐MSN	744.6	1.88	14.23

*Brunauer–Emmett–Teller (BET) method.

**Figure 3:**
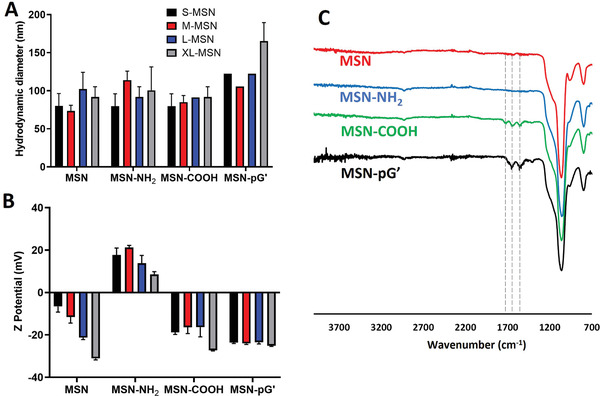
Characterization of prepared MSNs after each chemical modification step by DLS (upper left panel), Z potential (lower left panel), and FTIR spectra of the different chemical modification steps (M‐MSN materials shown as an example) (right panel). Data are Means ± SD (*n* = 3).

The prepared materials were then chemically modified by several stages: First, the MSNs were functionalized with amino groups, then, those amino groups were reacted with succinic anhydride to provide carboxylic acids on the MSN surface, which were used to covalently graft protein G’. The correct development of each stage was confirmed by several characterization techniques. DLS showed that no drastic change in particle size occurred at any stage of the process, confirming that the particles did not undergo aggregation by any of the modifications performed, although there was a slight increase in size for MSN‐pG’ particles compared to previous steps, especially for XL‐MSN‐pG’ (**Figure** **3**). Z potential measurements (Figure 3) showed the change from negative values in the newly synthesized material to positive values after amination (MSN‐NH_2_), returning to negative values after carboxylation (MSN‐COOH), and also negative final values for the protein G’‐grafted materials (MSN‐pG'). By means of Fourier transform infrared spectroscopy (FTIR), the characteristic bands of silica were observed in all the prepared materials (700–1300 cm^−1^). FTIR (Figure 3) also confirmed the presence of carboxylic acid groups in the carboxylated particles (band at 1720 cm^−1^), as well as amide group signals confirming the covalent anchoring of the succinic anhydride used to the amino groups of the particles (amide I and amide II bands at 1650 and 1560 cm^−1^ respectively). Finally, the disappearance of the carboxylic acid band and the increase in the relative intensity of the amide I and amide II signals confirm protein G’ binding. The amount of protein G’ grafted to each material was determined by thermogravimetric analysis (Table S1, Figure S2, Supporting Information), showing a mass percentage of 6.88% for S‐MSN, 7.89% for M‐MSN, 8.94% for L‐MSN, and 7.74% for XL‐MSN. These data confirm the initial part of our hypothesis, showing that the amount of protein G’ anchored to MSNs depends on the pore size of the nanoparticles, being largest for L‐MSN‐pG’.

### 2.2 Evaluation of IgG Capture Capacity Using Prepared Solutions of Known IgG Concentration

**Figure 4:**
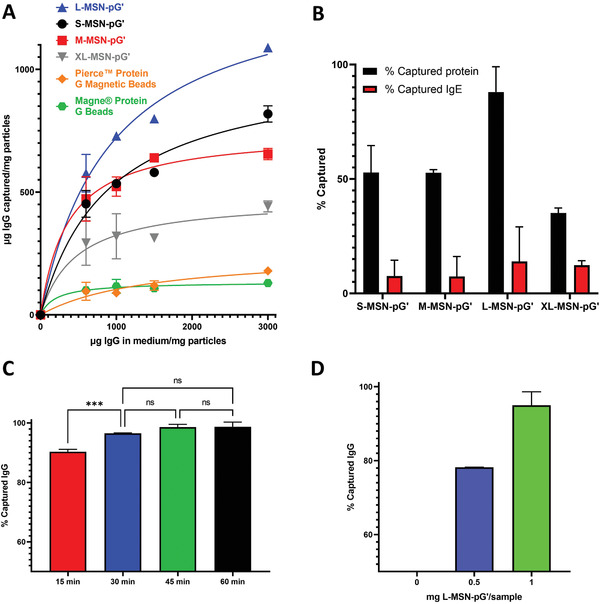
A) IgG capture studies by the prepared MSN‐pG' and by commercial IgG capture systems (Pierce Protein G Magnetic Beads and Magne Protein G Beads) using solutions of human IgG from a commercial source. B) IgG capture specificity study using IgG + IgE mixtures, evaluating total protein uptake by Nanodrop and IgE capture (FITC‐labeled) by fluorimetry. C) IgG uptake in a 1 mg mL^−1^ solution of IgG at different incubation times with L‐MSN‐pG'. D) IgG capture in 50 µL of a 10 mg mL^−1^ IgG solution using different amounts of L‐MSN‐pG'. Data are Means ± SD (*n* = 3). In panel A, the curves connecting experimental data points correspond to a non‐linear fit to a specific binding model using Graphpad Prism 9 Software. Statistical analysis performed by one‐way ANOVA for panel C, using Graphpad Prism 9 Software. ns *p* > 0.05; ***p < 0.001.

Once the desired nanoparticles were obtained, we evaluated their capacity for human IgG uptake. For this purpose, we dispersed different amounts of MSN‐pG’ in a solution containing 1 mg mL^−1^ of IgG. The same experiment was performed with MSN‐COOH (without protein G’) as a control. Furthermore, two commercial IgG capture systems were also evaluated to compare the performance of the prepared nanoparticles with state‐of‐the‐art materials (Pierce Protein G Magnetic Beads and Magne Protein G Beads). After half an hour of incubation at room temperature, the particles were removed by centrifugation and the amount of IgG remaining in the supernatant was quantified by spectrophotometry (using a Nanodrop equipment). The results obtained (Figure S3, Supporting Information) show that MSNs without protein G’ have a very low non‐selective IgG uptake (less than 15%), which does not seem to be dependent on particle concentration. On the other hand, all MSN‐pG’ showed human IgG withdrawal capacity, with an increase in the amount of IgG removed with increasing particle concentration. Moreover, among the four materials prepared, L‐MSN‐pG’ particles showed a significantly higher IgG uptake capacity compared to the other three materials, definitively confirming our starting hypothesis: by controlling the pore size of MSNs to which protein G’ is anchored, their IgG removal capacity can be optimized. Importantly, the IgG uptake capacity obtained with L‐MSN‐pG’ was also far superior to that of the two tested commercial systems (**Figure** **4**A), as well as being larger than what has been shown by other previously described systems.^[^ ^11^, ^12^, ^14^ ^]^

In order to estimate the theoretical binding capacity of the different pG’‐grafted materials prepared, the experimental IgG capture results were fitted to a specific binding model (Figure 4A, Table S3, Supporting Information) according to Equation (1).

(1) $$Y=B\max*X/\left(Kd+X\right)$$

where *B*max is the maximum theoretical binding and *Kd* is the equilibrium binding constant. The results from the fit (Table S3, Supporting Information) confirm that L‐MSN‐pG’ presents the largest theoretical IgG binding capacity, with a *B*max of 1387 µg of IgG per mg of nanoparticles. Regarding previous work with porous silica materials for IgG capture, one work reported protein A grafting to two different materials: one with 10 nm pores (in the mesoporosity range) and one with 100 nm pores.^[^ ^15^ ^]^ In that study, a higher IgG uptake capacity was observed in the 100 nm pore material, despite the fact that the amount of anchored protein A was higher in the 10 nm pore material. This fact is probably due to the difficult access of IgG to deep areas of the smaller pore size, since the particles used are 40 µm in diameter. As a precedent with mesoporous nanoparticles, Hu et al. described the preparation of magnetic particles coated with mesoporous silica to which protein G was covalently anchored.^[^ ^11^ ^]^ The starting material used had a pore size centered at 3.27 nm, and from this an expanded pore material (with a very wide distribution of pore diameters, between 5 and 100 nm) was also obtained. In that study, a higher IgG uptake capacity was observed in the expanded pore material, although the difference was modest (51 µg IgG mg^−1^ of expanded pore material vs 41 µg IgG mg^−1^ of 3.27 nm pore material). While this work is the first proof of concept that pore size expansion in mesoporous materials above 3.27 nm could improve IgG uptake capacity in protein G‐modified materials, no materials with different pore sizes were obtained to determine if there is an optimal size for material performance. Additionally, the IgG uptake capacity that they showed (51 µg IgG mg^−1^ of nanoparticles) would still be very insufficient for use in the clinical application that we propose. There are several reasons why the IgG capture capacity of our material largely overperforms the materials reported by Hu et al. First, the pore size distribution in the work by Hu et al. is very broad and TEM images show very little homogeneity in the porosity of the particles, which makes it impossible to determine the optimal pore size for IgG capture. On the other hand, the surface area of that expanded pore material (43.4 m^2^ g^−1^) is very small compared to other mesoporous materials, which may limit the amount of anchored protein and thus the IgG uptake capacity. These two limitations derive from the method used to produce the materials, based on obtaining materials with smaller pore sizes, which are subsequently expanded, resulting in a large heterogeneity in the final size of the pores obtained and a large decrease in the surface area of the material. In contrast, the method used in the current work allows a precise control of the pore size while maintaining a large surface area (>400 m^2^ g^−1^ for all the prepared MSNs). Other authors have also described the use of mesoporous alumina modified with dimercaptosuccinic acid to capture glycoproteins, showing an impressive IgG capture capacity of 2298.6 µg of IgG per mg of particles.^[^ ^16^ ^]^ However, as the interaction between IgG and that material is not fully specific, while it enables enriching a sample in IgG compared to other proteins (such as albumin), it would not allow to remove IgG from serum without also removing IgE, as we would need for in vitro allergy diagnosis. As this selective removal of IgG is key for our aimed application, the next step was to evaluate the specificity of IgG uptake using IgG + IgE mixtures. In this case, the concentration of IgG (1 mg mL^−1^) was much larger than the concentration of IgE (1 µg mL^−1^), as occurs in real clinical samples. To allow simultaneous detection of IgG and IgE capture, IgE was labeled with a fluorophore (fluorescein isothiocyanate, FITC). In that way, the amount of total protein in the supernatant after incubation with the nanoparticles (which will mostly correspond to IgG, as it constitutes 99.9% of the protein concentration) could be determined by spectrophotometry (using a Nanodrop equipment) while the amount of IgE (at much lower concentration) could be determined by fluorimetry. The results obtained from this experiment (Figure 4B) show that the resulting particles capture a high percentage of IgG while the removal of IgE was almost nonexistent. Given that L‐MSN‐pG’ had achieved a significantly larger IgG removal in previous experiments, this material was the one selected for further experiments aimed at optimizing the incubation conditions to ease later translation to clinical samples. Regarding the optimal incubation time of the nanoparticles with the sample, it was found that after 30 min an increase in incubation time (to 45 or 60 min) did not lead to a significant increase in the amount of IgG uptake (Figure 4C). Finally, the performance of L‐MSN‐pG’ in a clinically relevant IgG concentration (10 mg mL^−1^) was evaluated after an incubation of 30 min. The results showed that, while 0.5 mg of nanoparticles only removed 78.19 ± 0.05% of the IgG, 1 mg was enough to remove more than 94.99 ± 3.63% of the amount of IgG in solution (Figure 4D). After these preliminary experiments with solutions of known concentrations of immunoglobulins, the next step was to adapt the selected protocol to human serum samples, to determine the real‐scenario applicability of this optimized material (L‐MSN‐pG’).

### 2.3 Evaluation of IgG Removal Capacity Using Sera from Healthy Controls and Allergic Patients

**Figure 5:**
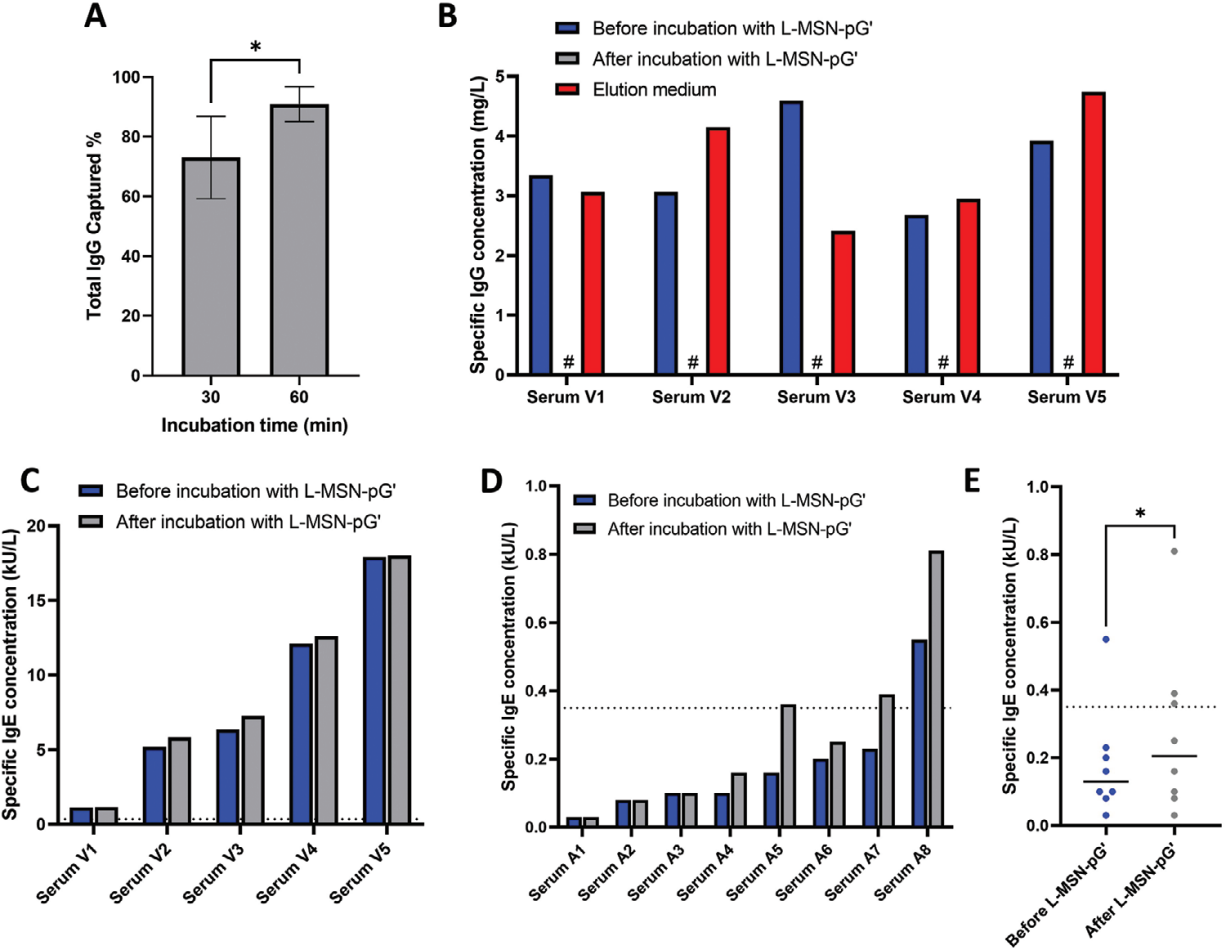
IgG capture studies by L‐MSN‐pG’ in human sera. A) Total IgG capture in sera from healthy controls (*n* = 3) after incubation for 30 or 60 min. B) Allergen‐specific IgG concentration before and after 1 h incubation with L‐MSN‐pG’ in sera from patients with confirmed allergy against *Vespula vulgaris* venom, as well as allergen‐specific IgG concentration in elution medium after release from L‐MSN‐pG’ (*n* = 5). C) Allergen‐specific IgE concentration before and after 1 h incubation with L‐MSN‐pG’ in sera from patients with confirmed allergy against *V. vulgaris* venom (*n* = 5). D,E) Amoxicillin‐specific IgE concentration before and after 1 h incubation with L‐MSN‐pG’ in sera from patients amoxicillin allergy confirmed by skin test or provocation (*n* = 8). Data in panel A are Means ± SD. Statistical analysis performed by Student's *t* test using Graphpad Prism 9 Software. *p < 0.05. #allergen‐specific IgG concentration in the sample was below detection limit for ImmunoCAP (Phadia).

The first step in the evaluation of L‐MSN‐pG’ in serum samples was to assess the total IgG removal capacity of this material in serum samples from healthy controls. For this, the amount determined in previous experiments (1 mg of L‐MSN‐pG’ dispersed in 25 µL of PBS) was mixed with 50 µL of serum from three different healthy controls. This mixture was stirred at room temperature for either 30 min or 1 h to determine the optimal conditions for IgG removal in serum, which was later measured with a commercially available “Human IgG ELISA Kit” (Sigma‐Aldrich), following the manufacturer's instructions. These two times of incubation were selected because even though previously half an hour was shown to be sufficient to remove over 90% of IgG in a 10 mg mL^−1^ IgG solution in PBS, in that sample there was nothing other than IgG to interact with the nanoparticles, while there is a wide array of different proteins (and other molecules) in a complex sample like serum, which could make it necessary for longer incubation times to achieve equivalent IgG removal. The results (**Figure** **5**A) show that, while after 30 min of incubation IgG removal was only partial (73.06 ± 13.77% of IgG removed as determined by ELISA), 1 h of incubation was enough to remove 90.93 ± 5.85% of total IgG from these sera samples. Then, the next step was to confirm the specificity of IgG capture in human serum samples. Since the main application of this work is aimed for in vitro allergy diagnostics, the key parameter determining the potential of L‐MSN‐pG’ is its capacity to remove IgG from serum without reducing the concentration of IgE. To test this, the same incubation conditions were used for sera from five patients allergic to the venom of common wasp (*Vespula vulgaris*). Then, the concentration of specific immunoglobulins against *V. vulgaris* venom (both IgG and IgE) were evaluated by a Phadia ImmunoCAP system, the most common technique used for in vitro allergy diagnosis in clinical laboratories. Additionally, since in some cases (both in allergy diagnosis and in other applications), the target immunoglobulin to be determined can also be IgG, the captured IgG was also eluted from the nanoparticles and allergen‐specific IgG was also quantified in this elution medium, to evaluate the possibility to recover captured IgG for further analysis. The results regarding allergen‐specific IgG (Figure 5B) show that, for all five sera from allergic patients analyzed, detectable allergen‐specific IgG levels were found before sample treatment with L‐MSN‐pG’ and below detection limit after sample treatment with L‐MSN‐pG’. Moreover, and although there was some variability among samples, the average recovery % of allergen‐specific IgG from the nanoparticles in elution medium was 105.15 ± 32.06% of the initially detected allergen‐specific IgG amount. These results show that L‐MSN‐pG’ not only constitutes an efficient system for IgG removal from human serum, but that the captured IgG can be later eluted from the material and used for further analysis. On the other hand, the results regarding *V. vulgaris*‐specific IgE (Figure 5C) showed no reduction in allergen‐specific IgE concentration for any of the five sera analyzed after treatment with L‐MSN‐pG’, highlighting the good specificity for IgG capture of the system. This constitutes an important advantage, as in a previous work it was determined that, after the three incubations needed to remove the IgG from human sera using a commercial protein G‐grafted beads, there was a 35% decrease in total IgE concentration.^[^ ^9^ ^]^ The good specificity together with the large IgG capture capacity of L‐MSN‐pG’ in human sera obtained under incubation conditions that would be easily adaptable in the clinical setting, highlight the great potential of this system for future translation in the context of in vitro diagnostics.

Finally, in order to prove the clinical utility of our proposed sample pretreatment system, and confirm our hypothesis that the presence of allergen‐specific IgG, in higher levels in serum, could impair the recognition of specific IgE, in lower level in serum, new experiments were carried out with sera from eight patients allergic to amoxicillin, which is challenging to diagnose with in vitro methods. These patients had been confirmed as allergic to amoxicillin by in vivo methods (either skin or provocation test). Prior to nanoparticle treatment, only one of the eight sera presented an amoxicillin‐specific IgE level (determined by Phadia ImmunoCAP) above the clinical threshold for a positive result (0.35 kU L^−1^). After treatment with L‐MSN‐pG’, there was an increase in the amoxicillin‐specific IgE detected by ImmunoCAP Phadia in five out of eight sera, with two of them going from a negative result (<0.35 kU L^−1^) to a positive one (>0.35 kU L^−1^) (Figure 5D). When comparing the amoxicillin‐specific IgE levels, a significant increase was found after L‐MSN‐pG’ treatment (paired *t* test, Figure 5E). These results show the clinical relevance of our results, as the positivity rate for allergen‐specific IgE went from 12.5% before nanoparticle treatment to 37.5% after treatment with L‐MSN‐pG’ for the eight allergic patients included in the experiment. This would enable confirming a larger population of allergic patients with in vitro methods without the need for more dangerous in vivo tests.

## 3 Conclusions

In this work, protein G’‐grafted mesoporous silica nanoparticles with four different pore sizes were prepared, characterized, and evaluated. The pore size of the nanoparticles was shown to influence the capacity of these nanoparticles to capture IgG, with large‐pore mesoporous particles outperforming the rest of the materials prepared. Then, a simple protocol was optimized to enable highly efficient and selective IgG removal from human serum, under conditions that would enable an easy adoption in the clinical setting. This practice was shown to be useful for both the purification of IgG and for the more feasible detection of low concentrated IgE in IgG‐free medium.

## 4 Experimental Section

### Preparation of Mesoporous Silica Nanoparticles Modified with Protein G’

Unless specified, all reagents were purchased from Sigma‐Aldrich (Spain) and were used without further purification. The mesoporous silica nanoparticles that will serve as the basis for the system were prepared by a previously described biphasic method based on the condensation of tetraethylorthosilicate (TEOS) in a biphasic water/cyclohexane system, using triethanolamine as the base and cetyltrimethylammonium chloride (CTAC) as the structure‐directing agent surfactant.^[^ ^17^ ^]^ The aqueous phase was composed of a mixture of 24 mL of a commercial aqueous solution of CTAC (25% w/v)), 0.18 g of triethanolamine and 36 mL of deionized water. The organic phase consists of 20 mL of a mixture of cyclohexane with TEOS. The concentration of TEOS depends on the material to be prepared: 20% for S‐MSN, 10% for M‐MSN, 5% for L‐MSN, and 2.5% for XL‐MSN. The synthesis reaction was carried out at 50 °C for 24 h. Subsequently, the surfactant was extracted by ion exchange with an ethanolic solution of ammonium nitrate (10 mg mL^−1^) at reflux for 1 h, followed by a second reflux for 2 h in an ethanolic solution of 12 mm HCl. Finally, the material was washed with ethanol three times to obtain the starting nanoparticles, already without surfactant. The surface (both the external surface of the particle and the exposed surface of the pores) was then functionalized with 3‐aminopropyltriethoxysilane (APTES) in toluene.^[^ ^18^ ^]^ This reaction was carried out in an inert atmosphere (using nitrogen), under reflux for 24 h, using 0.5 µL APTES/mg MSN. After washing the nanoparticles with toluene and ethanol, the conversion of the added amino groups into carboxylic acids was carried out.^[^ ^19^ ^]^ For this purpose, the aminated material was reacted with succinic anhydride (0.2 mg for each mg of MSN) in tetrahydrofuran (THF), under inert atmosphere and at room temperature for 24 h. Finally, after washing the material with THF and ethanol, protein G’ anchoring was produced by carbodiimide conjugation chemistry.^[^ ^20^ ^]^ For this purpose, carboxylic acids were activated on the surface of the particles by dispersing 5 mg of nanoparticles in 600 µL of buffer containing 2‐morpholinoethanesulfonic acid (MES, 50 mm, pH 5.5) with 1 mg of *N*‐hydroxysuccinimide (NHS) and 1 mg of *N*‐(3‐dimethylaminopropyl)‐*N*'‐ethylcarbodiimide hydrochloride (EDC). After stirring at room temperature for 1 h, the activated nanoparticles were collected by centrifugation and redispersed in 500 µL of phosphate buffered saline (PBS) with 1 mg of protein G’ in solution. After standing with agitation at room temperature for 24 h, the protein G’‐grafted MSNs were washed with PBS and redispersed again in PBS at a concentration of 10 or 40 mg mL^−1^ for further experiments.

### Characterization of Mesoporous Silica Nanoparticles

To confirm the correct preparation and chemical modification of the nanoparticles, their characterization was performed using multiple techniques. Dynamic light scattering (DLS) and Z‐potential measurements were performed with a Malvern Zetasizer Nano ZS90 instrument, checking both particle size and surface charge. The instrument used was equipped with a “red laser” (*ʎ* = 300 nm) and DLS measurements were performed with a detection angle of 90°, while the Smoluchowski approximation was used for Z‐potential measurements. For Fourier transform infrared spectroscopy (FTIR), a Jasco 4100 FT/IR machine equipped with an attenuated total reflectance (ATR) accessory was used. To check the morphology and the different pore size of the nanoparticles, the characterization of the nanoparticles was performed by transmission electron microscopy (TEM) on a Thermo Fisher Scientific Tecnai G2 20 Twin using copper grids of mesh size 200 coated with a Formvar‐Carbon film. Scanning electron microscopy (SEM) was carried out on a Thermo Fisher Quanta FEG 250. The nitrogen adsorption (in a Micromeritics ASAP 2020 unit) and thermogravimetry (in a MetTler Toledo TGA/DSC 1 unit) measurements were carried out at the Central Research Support Services (SCAI) of the University of Malaga (UMA).

### IgG Capture Experiments Using Immunoglobulin Solutions of Known Concentration

To evaluate the IgG removal capacity of the prepared nanoparticles, different amounts of nanoparticles (100, 300, or 500 µg) with or without anchored protein G’ were added to 300 µL of a 1 mg mL^−1^ dilution of human IgG in PBS. After incubation at room temperature with shaking for 30 min, the samples were centrifuged and the amount of IgG still in the supernatant was quantified spectrophotometrically on a Nanodrop 2000c (Thermo Scientific). Subsequently, an analogous experiment was carried out keeping the amount of nanoparticles constant (500 µg of particles for 300 µL of IgG solution) but evaluating the effect of incubation time (15, 30, 45, and 60 min) on IgG removal.

To evaluate the specificity of the system uptake, immunoglobulin uptake was evaluated in a mixture of human IgG and human IgE (which would be the analyte of interest in allergy diagnosis). In order to analyze IgE concentration in the solution with both immunoglobulins, fluorescent labeling of IgE was performed using FITC. IgE labeling was performed by incubating 25 µg of human IgE together with 2 µg of FITC in 100 µL of carbonate buffer (pH = 10.2). After stirring at room temperature for 1 h, labeled IgE was purified using an Amicon system (30 kDa). Using this labeled IgE and unlabeled IgG, IgG + IgE‐FITC mixture was prepared in PBS (1 mg mL^−1^ IgG, 1 µg mL^−1^ IgE‐FITC). After incubating the nanoparticles with the IgG + IgE‐FITC dilution for half an hour at room temperature (500 µg of particles for 300 µL of IgG + IgE‐FITC mixture), the samples were centrifuged, and the supernatants were analyzed by Nanodrop spectrophotometry to assess the total concentration of protein not captured by the nanoparticles. Subsequently, a 1:3 dilution of the supernatants with PBS was performed to measure IgE concentration using a fluorometer.

Finally, to evaluate IgG removal under clinically relevant concentrations, 50 µL of a 10 mg mL^−1^ IgG solution in PBS were incubated with L‐MSN‐pG’ by adding 12.5 or 25 µL of a 40 mg mL^−1^ nanoparticle suspension (particle dose 0.5 or 1 mg) and stirring at room temperature for 30 min. After centrifugation, IgG concentration in the supernatant was quantified spectrophotometrically on a Nanodrop 2000c (Thermo Scientific).

### Evaluation of IgG Capture Capacity and Specificity Using Human Sera

Sera from both healthy controls (*n* = 3) and patients allergic to common wasp (*V. vulgaris*) venom with confirmed allergen‐specific IgE by ImmunoCAP (*n* = 5) were used to evaluate the utility of L‐MSN‐pG’ for the removal of IgG in clinical samples. The study was conducted according to the Declaration of Helsinki principles and was approved by the Provincial Ethics Committee of Malaga (approval no. PIN‐0113‐2020). All subjects included in the study were informed orally and signed the corresponding informed consent.

First, to evaluate the IgG removal capacity of L‐MSN‐pG’ in human serum, 1 mg of these nanoparticles dispersed in 25 µL of PBS were added to 50 µL of serum from healthy controls (*n* = 3). After incubation at room temperature with shaking for 30 or 60 min, the samples were centrifuged and the amount of IgG still in the supernatant was quantified by a commercial “Human IgG ELISA Kit” (Sigma‐Aldrich, Product RAB0001, Lot # 0601I225), following the manufacturer's instructions. Control measurements were also carried out with a mixture of 25 µL of PBS (without nanoparticles) and 50 µL of serum to quantify the total IgG amount in the sample before incubation with L‐MSN‐pG’, enabling the determination of the % of IgG captured.

Then, to evaluate the both the allergen‐specific IgG removal capacity of L‐MSN‐pG’ in sera from allergic patients, as well as to determine the specificity of allergen‐specific IgG capture compared to allergen‐specific IgE capture, 2 mg of these nanoparticles dispersed in 50 µL of PBS were added to 100 µL of serum from patients with confirmed allergy against common wasp (*V. vulgaris*) venom (*n* = 5) or against amoxicillin (*n* = 8). After incubation at room temperature with shaking for 60 min, the samples were centrifuged and the amount of both allergen‐specific IgG and allergen‐specific IgE still in the supernatant were quantified ImmunoCAP (Phadia). Control measurements were also carried out with a mixture of 50 µL of PBS (without nanoparticles) and 100 µL of serum to quantify the amount of allergen‐specific IgG (*V. vulgaris*) and allergen‐specific IgE (*V. vulgaris* or amoxicillin) in the sample before incubation with L‐MSN‐pG’. The captured IgG was also eluted from the nanoparticles by dispersing the nanoparticle pellet (with 20 µL of residual liquid from the previous incubation) in 115 µL of “Pierce IgG Elution Buffer” (ThermoFisher) using a sonication bath. Then, the sample was centrifuged, and the supernatant was transferred to a new tube and mixed with 15 µL of “Neutralization Buffer” (ThermoFisher). After this, the concentration of specific IgG in this elution medium was also determined by ImmunoCAP (Phadia).

## Conflict of Interest

J.L.P., T.D.F., M.I.M., C.M., and M.J.T. have a patent application (P202230701) pending, which is related to the work reported in this article. The rest of the authors declare no conflict of interest.

## Supporting information

Supporting Information

## Data Availability

The data that support the findings of this study are available from the corresponding author upon reasonable request.
