# Supplementary material for: Influence of Pore Size in Protein G'‐Grafted Mesoporous Silica Nanoparticles as a Serum Pretreatment System for In Vitro Allergy Diagnosis
Source: Adv Healthc Mater. 2023 Mar 8;12(15):2203321. doi: 10.1002/adhm.202203321 (PMC11468951; doi:10.1002/adhm.202203321)
Supplement: Supplementary file 1 — Supporting Information [file ADHM-12-2203321-s001.pdf]

ADVANCED
HEALTHCARE
MATERIALS

Supporting Information

for *Adv. Healthcare Mater.*, DOI 10.1002/adhm.202203321

Influence of Pore Size in Protein G'-Grafted Mesoporous Silica Nanoparticles as a Serum Pretreatment System for In Vitro Allergy Diagnosis

Juan L. Paris, Cristina Monío, Ana M. Pérez-Moreno, Raquel Jurado-Escobar, Gador Bogas, Tahía D. Fernández, María I. Montañez, Cristobalina Mayorga* and María J. Torres**

SUPPORTING INFORMATION

Influence of pore size in protein G'-grafted mesoporous silica nanoparticles as a serum pretreatment system for *in vitro* allergy diagnosis.

Juan L. Paris, Cristina Monío, Ana M. Pérez-Moreno, Raquel Jurado-Escobar, Tahía D. Fernández, María I. Montañez, Cristobalina Mayorga, María J. Torres

Table S1. Organic matter % of S-MSN, M-MSN, L-MSN and XL-MSN particles, at two chemical modification steps: MSN-COOH or MSN-pG', determined by thermogravimetric analysis. Estimation of Protein G' mass %.

Sample	Weight loss % (100-600°C)		Protein G' mass % (Δ weight loss %)
	MSN-COOH	MSN-pG'	
S-MSN	18.89	25.77	6.88
M-MSN	19.72	27.61	7.89
L-MSN	19.99	28.93	8.94
XL-MSN	24.70	32.44	7.74

Table S2. IgG capture capacity of different commercial systems based on their manufacturer's descriptions.

**Commercial system**	**IgG capture capacity**
SureBeads™ (Bio-Rad)	≥6 µg IgG/mg particles
Pierce™ Protein G Magnetic Beads (ThermoFisher)	≥60 µg IgG/mg particles
Absolute Mag™ Protein G Magnetic Particles (Creative Diagnostics)	>60 µg IgG/mg particles
Sera-Mag SpeedBeads Protein A/G (cytiva)	55 - 85 µg IgG/mg particles
Magne™ Protein G Beads (Promega)	125 µg IgG µg IgG/mg particles

Table S3. Fit parameters of IgG binding capacity to a specific binding model.

	**S-MSN-pG'**	**M-MSN-pG'**	**L-MSN-pG'**	**XL-MSN-pG'**
Bmax	1022	747.8	1387	475.7
Kd	892.0	358.5	923.2	468.3
R^2	0.9823	0.9920	0.9917	0.9623

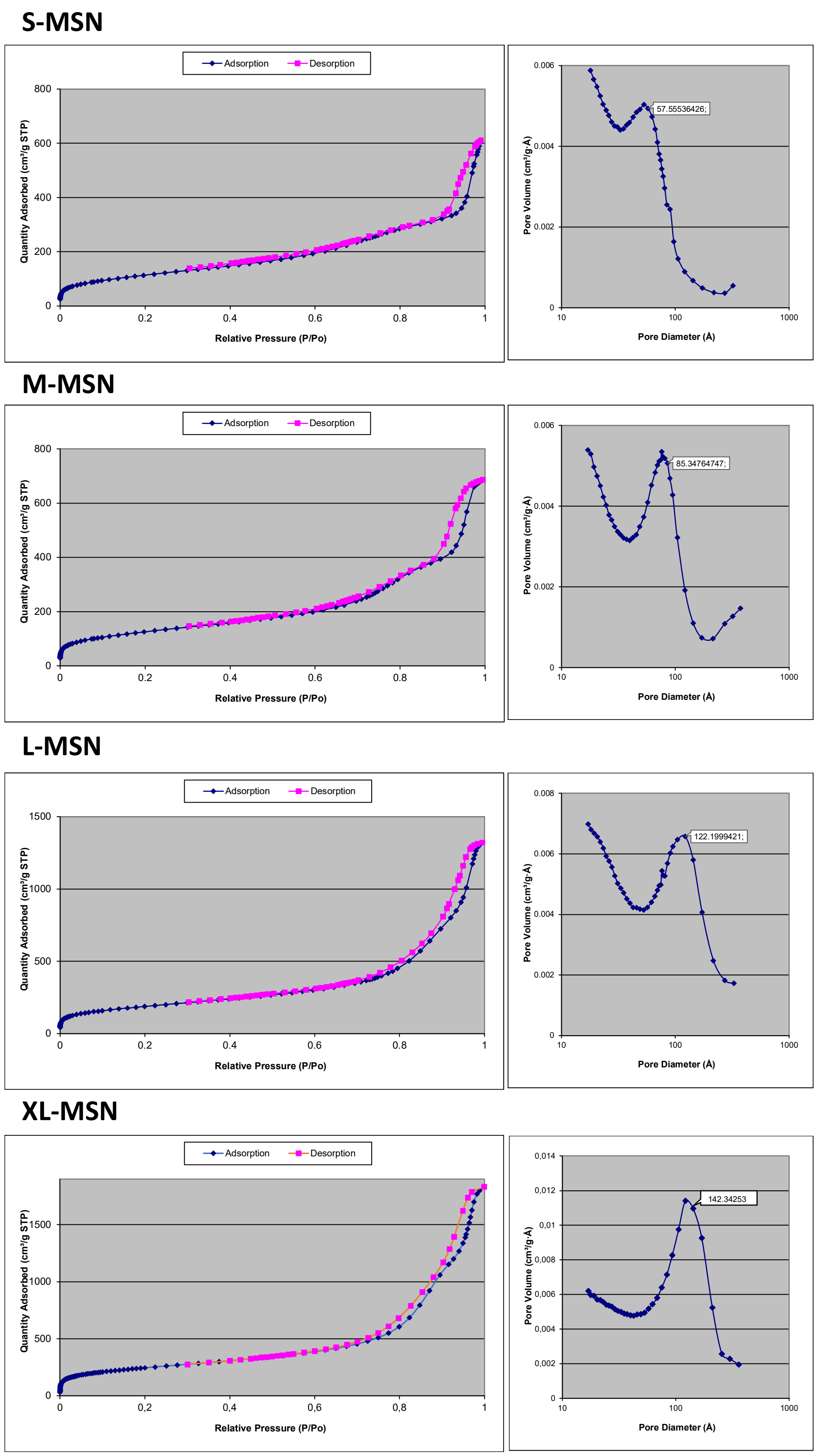

Figure S1. Characterization of prepared MSNs by N_2 adsorption, showing N_2 adsorption-desorption isotherms (left) and pore size distribution curve based on the Brunauer-Emmett-Teller (BET) method. Results for (top to bottom): S-MSN, M-MSN, L-MSN and XL-MSN.

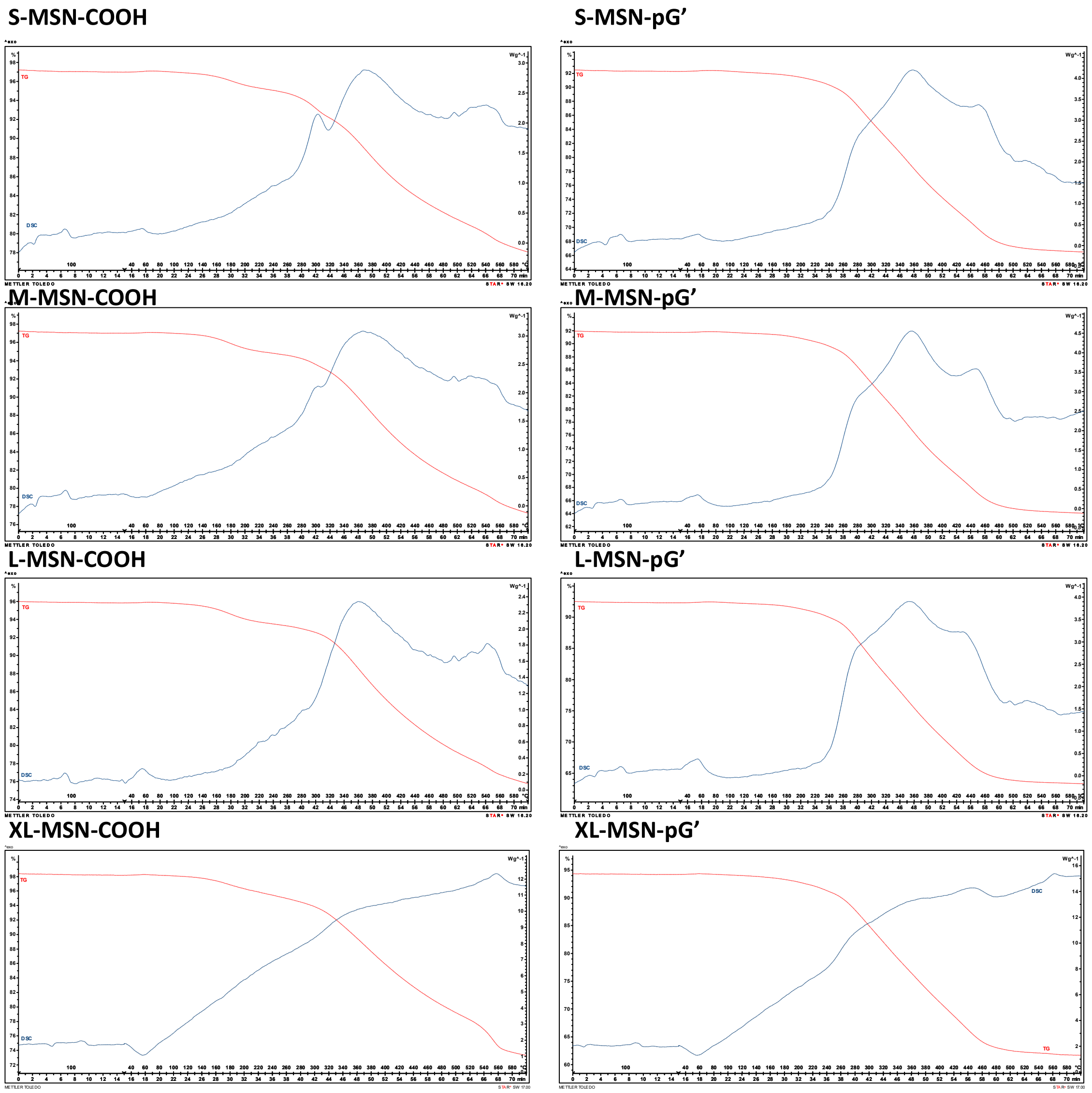

Figure S2. Characterization of prepared MSNs by thermogravimetric analysis. Results for (top to bottom) S-MSN, M-MSN, L-MSN and XL-MSN, at two different chemical modification steps: MSN-COOH (left) or MSN-pG’ (right).

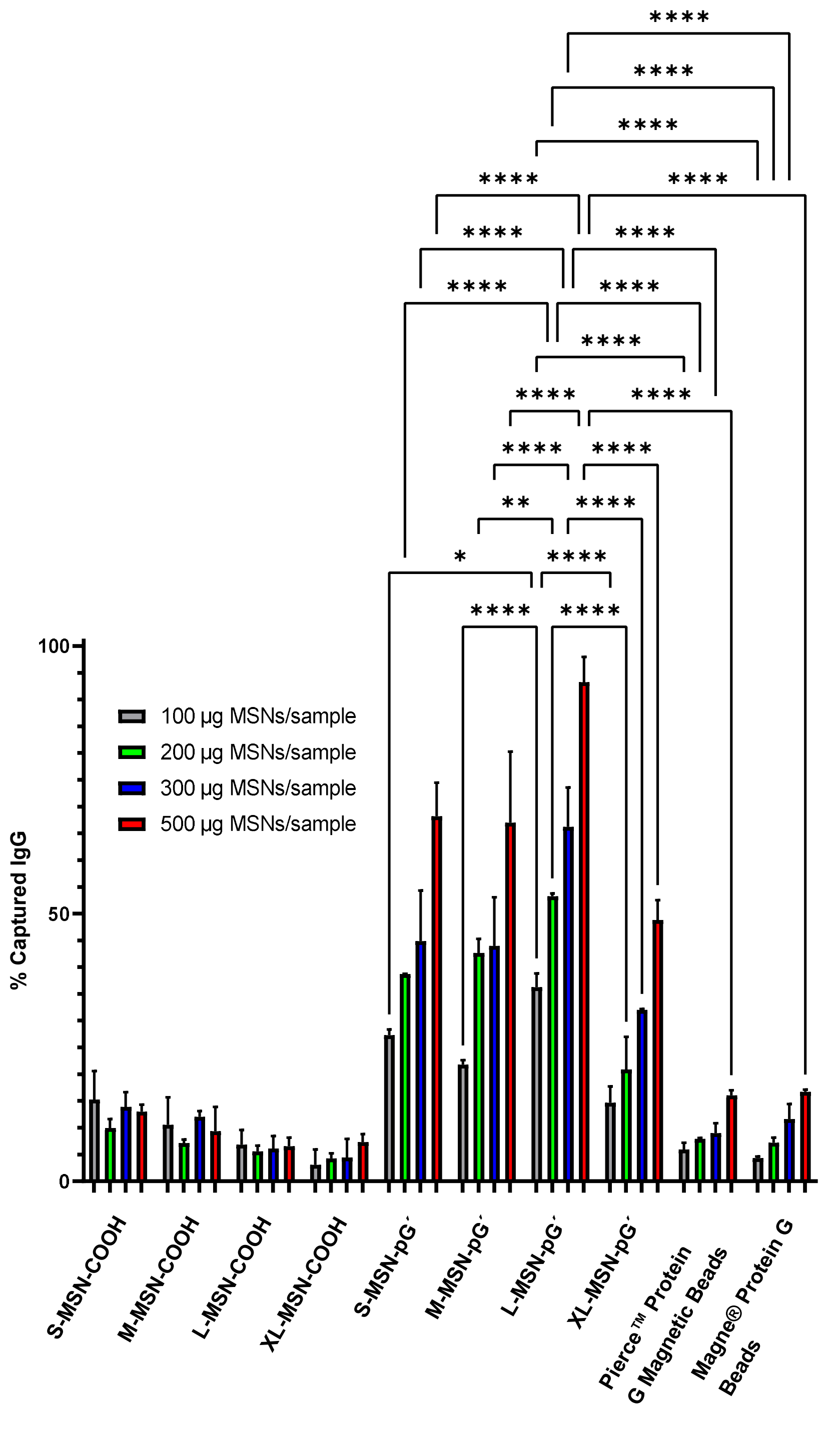

Figure S3. IgG capture studies by the prepared materials in solutions of known IgG concentration from a commercial source. IgG uptake in a 1 mg/mL IgG solution using different amounts of MSN-COOH, MSN-pG' and commercial IgG capture systems (Pierce™ Protein G Magnetic Beads and Magne™ Protein G Beads). Data are Means ± SD (n=3). Statistical analysis performed by two-way ANOVA using Graphpad Prism 9 Software. *$p<0.05$; **$p<0.01$; ***$p<0.001$; ****$p<0.0001$.
